# Analysis of Porcine Model of Fecal-Induced Peritonitis Reveals the Tropism of Blood Microbiome

**DOI:** 10.3389/fcimb.2021.676650

**Published:** 2021-08-30

**Authors:** Hwi Hyun, Min Seok Lee, Inwon Park, Hwa Soo Ko, Seongmin Yun, Dong-Hyun Jang, Seonghye Kim, Hajin Kim, Joo H. Kang, Jae Hyuk Lee, Taejoon Kwon

**Affiliations:** ^1^Department of Biomedical Engineering, College of Information and Biotechnology, Ulsan National Institute of Science and Technology (UNIST), Ulsan, South Korea; ^2^Department of Emergency Medicine, Seoul National University Bundang Hospital, Seongnam-si, South Korea; ^3^Center for Genomic Integrity, Institute for Basic Science, Ulsan, South Korea

**Keywords:** blood microbiome, peritonitis, porcine (pig) model, bloodstream infection (BSI), dysbiosis

## Abstract

Recent studies have suggested the existence of a blood microbiome in the healthy host. However, changes in the blood microbiome upon bloodstream infection are not known. Here, we analyzed the dynamics of the blood microbiome in a porcine model of polymicrobial bacteremia induced by fecal peritonitis. Surprisingly, we detected bacterial populations in the bloodstream even before the infection, and these populations were maintained over time. The native blood microbiome was notably taxonomically different from the fecal microbiome that was used to induce peritonitis, reflecting microbial tropism for the blood. Although the population composition after the infection was similar to that of the native blood microbiome, new bacterial strains entered the bloodstream upon peritonitis induction as clinical symptoms relevant to sepsis developed. This indicates that the bacteria detected in the blood before peritonitis induction were derived from the blood rather than a contamination. Comparison of the functional pathways enriched in the blood and fecal microbiomes revealed that communication and stress management pathways are essential for the survival of the blood microbiome.

## Introduction

Bloodstream infection (BSI) is defined as a medical condition, in which viable bacteria or fungi are present in the bloodstream (Viscoli, 2016). BSI is a major threat to human health, as it can cause sepsis and organ dysfunction (Cecconi et al., 2018). A survey of the incidence of BSI in America and Europe during the years 1974-2008 reported rates between 80 and 189 per 100,000 individuals per year; this number has increased in recent years (Laupland, 2013; Laupland et al., 2020). Furthermore, many cases progress to critical conditions. BSI is estimated to cause 79,000–94,000 deaths per year in North America and 157,000 deaths per year in Europe (Goto and Al-Hasan, 2013).

Blood culture is a well-established method of detecting BSI, but the blood culture findings are not always clinically relevant. According to a recent study, 42.6% of 2,659 patients with suspected sepsis had a positive blood culture result, whereas the remaining 1,526 patients (56.4%) were blood culture-negative (Nannan Panday et al., 2019). False positives caused by contamination are a concern when testing for BSI. For example, according to some studies, only 51% of blood culture-positive samples represent actual BSI, 41% are a result of contamination, and 8% have unknown clinical significance (Weinstein et al., 1997; Pien et al., 2010). The expected sensitivity and specificity of blood culture findings vary depending on the experimental conditions, including collection time, skin preparation prior to sampling, sampling site, and sample volume (Lamy et al., 2016).

High-throughput sequencing is an alternative technique for detecting microbes in the blood, even without culturing (Grumaz et al., 2016). However, this highly sensitive method raises some questions about BSI, i.e., on the existence of the blood microbiome [reviewed in (Castillo et al., 2019)]. Although the bloodstream is considered to be a sterile environment, recent evidence suggests that it may contain bacteria (or a microbiome), which may also colonize other organs. According to a 1969 study, metabolically active bacteria might be present in the blood (Tedeschi et al., 1969), and recent studies propose that bacteria may use the bloodstream as a transport system. For example, bacteria have been identified in the blood and adipose tissue samples from patients with type 2 diabetes (Massier et al., 2020), and in the liver of patients with non-alcoholic fatty liver disease (Sookoian et al., 2020). Furthermore, *Porphyromonas gingivalis* derived from chronic periodontitis is thought to contribute to Alzheimer’s disease (Dominy et al., 2019), leading to a speculation that the human microbiome can disseminate to other organs *via* the bloodstream.

Nonetheless, most of the above studies analyzed the blood microbiome at a single time point, making it difficult to rule out the possibility of contamination. Suppose the microbiota is stably maintained in the bloodstream. In that case, it should be detectable over time, like other microbiomes in the body. Concordance between data for different time points could support the existence of the blood microbiome. However, this type of data is difficult to collect for human samples. Furthermore, these data should be assessed alongside blood culture results. Even in cases of BSI, a limited number of bacterial cells are present in the blood (approximately 0.1 to 100 cells per 1 mL of infected blood) (Lamy et al., 2016). If in fact the blood microbiome exists, it is questionable why these bacteria are not detected by blood culture. Hence, a controlled experimental environment is required to evaluate the relationship between BSI and the blood microbiome.

Recently, we have developed a porcine model of fecal-induced peritonitis (Park et al., 2019). In the model, we observed symptoms of organ dysfunction approximately 7 hours (median) after introducing feces into the pig abdomen. The likelihood of detecting bacteria by blood culture also gradually increased after the induction. Here, we used the same blood samples as those obtained in a previously reported study of the porcine fecal-induced peritonitis model (Park et al., 2019) to investigate the role of the blood microbiome in bacteremia. Surprisingly, we detected many bacterial cells in the pig blood even before fecal induction. These bacteria likely constitute the blood microbiome. In addition, the bacterial species identified by blood culture were not the dominant species detected by 16S rRNA gene sequencing, although their increases over time tended to be the similar over time. The data presented herein provide new insights into the relationship between BSI and the blood microbiome.

## Materials and Methods

### Animal Experiments

Six domestic pigs (*Sus scrofa domesticus*), weighing approximately 45–55 kg each, were used, as described previously (Park et al., 2019). Autologous feces were collected 1 day before the experiment and preserved overnight at room temperature. The pigs were anesthetized by an intramuscular administration of zolazepam (zoletil, 5 mg/kg; Virbac, Carros, France). The animals were scrubbed with povidone-iodine soap and shaved, and monitoring devices, including an electrocardiograph, pulse oximeter, and a temperature probe, were attached. Then, the animals were intubated using an endotracheal tube and connected to a mechanical ventilator (Drager Fabius GS, Lubeck, Germany) providing an inhalation agent (sevoflurane; Baxter Inc., Deerfield, IL) to maintain adequate ventilation with anesthesia. A sterile surgical drape for the abdomen was applied after meticulous dressing with povidone-iodine. Under the guidance of ultrasound, two 6-Fr arterial catheters (Merit Medical, South Jordan, UT) were inserted into the two femoral arteries to allow invasive blood pressure monitoring and repetitive blood sampling for blood culture. The feces (1 g/kg) that had been collected the previous day were diluted in 5% dextrose saline (10 g/dL) and warmed at 37°C for 1 hour in a water bath. A midline surgical incision was made in the abdomen, and the feces were introduced into the abdominal cavity. Using the aseptic technique, blood samples (10 mL) were abstracted *via* the femoral arterial catheter at 1 or 2 hour intervals, and split between a bottle containing DNA/RNA Shield reagent (Cat # R1150; Zymo Research, Irvine, CA), and a pair of bottles for aerobe and anaerobe blood culture (BD BACTEC, Becton Dickinson, NJ). The pairs of blood culture bottles were then immediately placed in a blood culture system (BD BACTEC). The final microbiological report on blood culture findings was obtained 5 days after the experiment from the Department of Laboratory Medicine (Seoul National University Hospital, Seoul, Republic of Korea). After the induction with feces, the pigs were monitored for 12 hours. The primary goal was to maintain the mean arterial pressure over 65 mmHg, with maximal fluid (balanced crystalloid solution) and vasopressor (norepinephrine, vasopressin, and epinephrine) support. All procedures were approved by SNUBH IACUC (BA1804-246/040-01).

### Blood Microbiome Capture

Mannose-binding lectin (MBL)-coated magnetic nanoparticles (MNPs; 2 mg/mL) were added to 3 mL of the blood–DNA/RNA Shield solution (Cat # R1150, Zymo Research), and the samples were incubated for 20 min at room temperature. Captured bacteria were harvested using N52 magnets (BYO88-N52; KJ Magnetics, Pipersville, PA) and washed with PBS to remove other blood components. The captured bacteria were stored in the DNA/RNA Shield reagent at –20°C before use.

### Fecal Sample Preparation for Microbiome Analysis

Autologous feces (3 mL) from each pig were diluted in dextrose saline (10 g/mL) in a tube of DNA/RNA Shield reagent (Cat # R1150, Zymo Research) and stored at –20°C before analysis. Total genomic DNA was extracted using the MoBio PowerFecal^®^ DNA Isolation Kit (Cat # 12830-50, MO BIO Laboratories, Carlsbad, CA) and FastPrep-24™ (MP Biomedicals, LLC, Irvine, CA).

### DNA Extraction From ZymoBIOMICS Microbial Community Standard

Total genomic DNA was extracted from 20 μL ZymoBIOMICS Microbial Community Standard (Cat # D6300, Zymo Research) using the ZymoBIOMICS DNA Miniprep Kit (Cat # D4300, Zymo Research).

### Library Preparation

Beads with the captured bacteria were incubated at 37°C for 1 hour in a solution of lysozyme (10 mg/mL; Cat # 10837059001; Roche, Basel, Switzerland) in 10 mM Tris-HCl (pH 8.0). The beads were then transferred to lysis buffer (10 mM Tris-HCl [pH 7.4], 10 mM EDTA, and 2% SDS) containing 0.5 mg/mL proteinase K (Cat # B-2008; GeNetBio, Daejeon, Republic of Korea), and incubated overnight at 37°C. Genomic DNA was extracted from cell lysates by adding a phenol–chloroform–isoamyl alcohol mixture, followed by overnight incubation at –20°C. Next, DNA was precipitated with 0.6 volumes of isopropanol and 0.1 volume of 3 M sodium acetate (Cat # SR2006-050-55; Biosesang, Seongnam, Republic of Korea). After washing with 70% ethanol, the pellet was resuspended in 100 μL TE buffer for 30 min at 37°C. Then, 1 μL RNase A (Cat # B-2007, GeNetBio) was added, and the samples were incubated for 30 min at 37°C to remove RNA contamination. DNA was purified using Zymo DNA Clean & Concentrator-5 Kit (Cat # D4014, Zymo Research) and eluted in 20 μL RNase-free water.

### Sequencing of the V34 Region of the 16S rRNA Gene

Because all samples (except the fecal samples) contained a limited amount of DNA, RT-Q-PCR was performed using MIC qPCR (BioMolecular Systems, Upper Coomera, QLD) to determine the number of amplification cycles before saturation (typically, 20–28 cycles). After an initial PCR with V34 primers, eight cycles of adapter-ligation PCR were performed. The final library was sequenced using Illumina MiSeq (Illumina, San Diego, CA) in a 2 × 250 bp configuration.

### Fluorescence *In Situ* Hybridization (FISH) for Native Blood Microbiome

The bacterial samples enriched by the human recombinant mannose-binding lectin(hrMBL)-coated magnetic nanoparticles (MNPs) were fluorescently stained with 4′,6-diamidino-2-phenylindole (DAPI), and Cy3-labeled DNA FISH probes (5’-CTTGTACACACCGCCCGTCACACC-3’) targeting universal bacteria-specific ribosomal RNA sequences for quantitating the blood microbiome in the control blood sample from a porcine model. To magnetically concentrate the sample, we adopted a sinusoidal-shaped polydimethylsiloxane (PDMS) microfluidic device (300 µm × 200 µm; width × height) to capture the MNP-bound bacterial cells by locating a magnet (BYO88-N52, KJ Magnetics, PA, USA) underneath the device. Then, FISH reagents were sequentially injected by a syringe pump at a flow rate of 10 μL/min into the microfluidic channel. For fixation and permeabilization, the bacterial samples magnetically sequestered in the device were treated in the order of 24% ethanol(v/v) in 1X Tris-Buffered Saline (TBST) with 5 mM CaCl_2_ (5 min), washing buffer (3 min), 99% methanol (5 min), and washing buffer (3 min). 1X TBST supplemented with 5 mM CaCl_2_ was used for the washing buffer. Then, DNA FISH probes suspended in a hybridization solution (0.5 µM) were incubated with the samples (1 hour at 45°C), followed by a DAPI staining (30 min) and washing by 2X Saline Sodium Citrate Buffer (SSC) buffer. Finally, FISH images were obtained with a confocal microscope (LSM 780 Configuration 16 NLO multi-photon confocal microscope, Zeiss, Germany) with DAPI and Cy3 fluorescence filter sets.

### Taxonomic Analysis

PCR amplicons obtained using the Illumina V34 PCR primers were selected using ipcress (provided in exonerate version 2.2) after concatenating paired-end reads (Slater and Birney, 2005). Next, taxonomic information was assigned to each paired read using the Ribosomal Database Project (RDP) classifier (version 2.11) (Wang et al., 2007) and the RDP database (release 11.5) (Cole et al., 2014), at a confidence score > 0.8. The cut-off was determined by sequencing of the ZymoBIOMICS Microbial Community Standard, as described in **Supplementary Figure 1**. Operational taxonomic units (OTUs) were defined by clustering concatenated V34 amplicons at 99% identity using VSEARCH (version 2.13.6) (Rognes et al., 2016) (**Supplementary Data 1**). Ambiguous clusters with > 5% of reads assigned to a different genus than the seed genus assigned to the same cluster were removed. OTUs with low abundance, i.e., accounting for less than 0.4167% of all OTUs in all analyzed samples (N=6), were excluded from the taxonomic analysis. After defining genus-level clusters, additional taxonomic levels (from the genus to the phylum, including the family, order, and class) were defined. OTUs that were not present at the initial time point (i.e., before the induction of fecal peritonitis) were identified after selecting clusters with no reads from the initial samples. Changes in the initial microbiome after fecal induction were analyzed by using SourceTracker2 with default options (Knights et al., 2011). The proportions of bacterial species from unknown sources for the first three time points (T02, T04, and T05) and the last three time points (T10, T11, and T12) were compared using Wilcoxon test.

### Pathway Analysis Using Phylogenetic Investigation of Communities by Reconstruction of Unobserved States (PICRUSt2)

Differences in biological pathways enriched in different microbiome populations were investigated using PICRUSt2 (version 2.2.0-b) (Douglas et al., 2020), by determining the relative enrichment of biological pathways in each sample. Kyoto Encyclopedia of Genes and Genomes (KEGG) orthologs and pathways inferred at the genus level (99% OTUs) were entered into the PICRUSt2 metagenome pipeline. Results of the KEGG ortholog analysis were assigned to KEGG pathways based on PICRUSt2-appended default files. KEGG ortholog enrichment values without pathway information were discarded, and the remaining values were summed. If KEGG orthologs belonged to more than two KEGG pathways, they were added in individually. The proportion of each pathway between the blood microbiome over time and the fecal microbiome used to induce peritonitis were compared using one-sample Wilcoxon test.

## Results

### Blood Microbiome in the Porcine Model Before Peritonitis Induction

Because the number of bacteria in the blood is estimated to be 0.1–100 colony-forming units (CFUs)/mL, even in BSI (Lamy et al., 2016), we expected to observe similar numbers for the blood microbiome in the porcine bacteremia model. First, we enriched bacteria present in 3 mL blood using opsonin-coated magnetic beads (Kang et al., 2014). We then extracted the genomic DNA and performed 16S rRNA gene sequencing using Illumina V34 primers. For the 12 time points analyzed, we obtained 116,062.39 paired reads, on average, per sample from each of the six animals (median, 102,654 reads; minimum, 38,160 reads; maximum, 333,130 reads; all reads are available at the ENA under the accession ID PRJEB39083). We next clustered the reads to define OTUs and performed taxonomic analysis (from the phylum to genus level) using the RDP classifier (Wang et al., 2007) (See *Materials and Methods* for details; **Supplementary Data 2** for genus and **Supplementary Data 3** for phylum).

**Figure 1:**
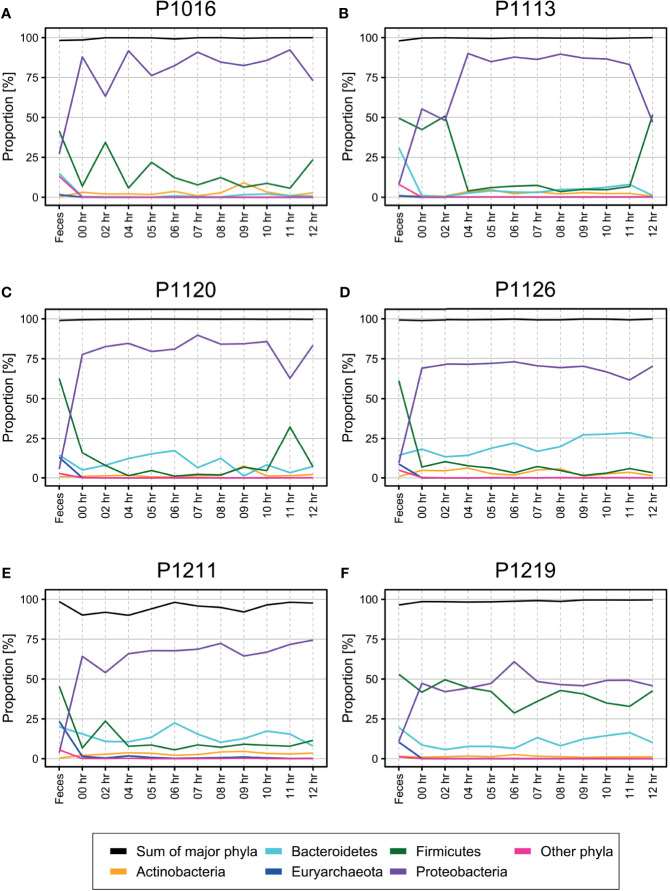
**(A–F)** Percentage of operational taxonomic units (OTUs) from each phylum identified in the blood of pigs with fecal-induced peritonitis. The proportion of each phylum in the blood microbiome was maintained throughout the induction period, which was different from the observations for the fecal microbiome. Although *Proteobacteria* was the major phylum identified in the blood, its percentage contribution varied in each of the six animals tested. The proportions at the genus level are shown in **Supplementary Figure 2**.

Surprisingly, we observed many bacterial species at the initial time point, i.e., even before the induction of fecal peritonitis. Furthermore, the bacterial populations did not change much over 12 h (**Figure 1**, phylum level; **Supplementary Figure 2**, genus level). *Firmicutes* and *Bacteroidetes* were the most abundant phyla in the fecal microbiome, while *Proteobacteria* was the most abundant phylum in the blood. The bacterial composition of four out of six animals tested (P1120, P1126, P1211, and P1219; **Figure 1**) was relatively constant, with few perturbations (e.g., at 11 h post-induction in P1120 and 6 h post-induction in P1219). The microbiome composition in the two other animals (P1016 and P1103) showed some fluctuations during the early induction stage, but it stabilized 4 h after the induction.

To confirm the presence of microbiome before the fecal induction, we also performed a DNA-FISH experiment using the bead-captured bacterial cells and a universal probe (5′-CTTGTACACACCGCCCGTCACACC-3′), which hybridizes to the 16S rRNA sequences of 98% bacterial species available in the Genome Taxonomy Database (GTDB; release 89) (Parks et al., 2018) (**Supplementary Figure 3**). Although the blood culture findings for all samples were negative, we verified that the blood samples obtained before peritonitis induction contained bacteria as observed in the FISH images. These observations suggest the presence of the blood microbiome in the porcine bacteremia model even before obtaining a positive blood culture result.

### Characterization of the Initial Blood Microbiome in the Porcine Model

It is possible that the microbiome detected in the blood samples may have reflected the native blood microbiome or contamination of the arterial catheter used for sampling or contamination by the skin microbiome, as discussed previously (Trautner and Darouiche, 2004; Horiba et al., 2018; Okuda et al., 2018). Further, because the number of bacterial cells in the samples was small, the observed microbiome could represent an uncontrollable low biomass contamination from an unknown source, known as the “KitOme” (Stinson et al., 2019). If the majority of identified bacteria came from an accidental contamination, one would expect to not see any discernible patterns in the blood microbiome profiles. However, if the bacteria were blood microbiome related to peritonitis induction, we would detect new types of bacteria that entered the bloodstream.

**Figure 2:**
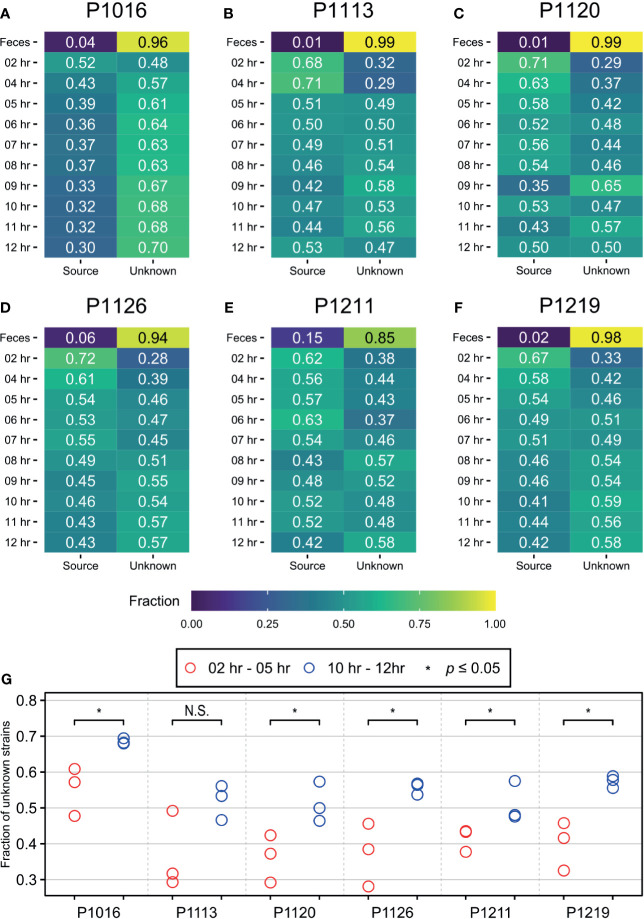
SourceTracker2 analysis indicating that the population of novel bacterial strains not present at the initial time point increased throughout the induction period. **(A–F)** The microbiome observed at the initial time point is designated as the “source”. The proportions of species that originated from the source were then tracked throughout the induction period. The proportion of unknown strains increased gradually until approximately 7 to 8 h after the induction before being maintained. **(G)** Wilcoxon test was used to determine the significance between first three time points (2, 4, 5 hours after induction) and the last three time points (10, 11, 12 hours after induction), and meaningful increase of an “unknown” microbiome was observed in five of six animals. * denotes the p-value of the Wilcoxon test is less than 0.05. N.S., Not Significant.

To distinguish between the two possibilities, we used SourceTracker2 (Knights et al., 2011) and compared the blood microbiomes before and after peritonitis induction (**Figure 2**; **Supplementary Data 4**). We assumed that the initial time point would be the “sink” for “the native blood microbiome” and attempted to identify their trends over time. In all animals, the bacterial population detected at the initial time point (sink) decreased gradually until approximately 6–8 hours after peritonitis induction and then remained constant until 12 hours after the induction. This trend matched the physiological symptoms of sepsis (Park et al., 2019). Therefore, we speculated that the blood microbiome was altered approximately 6–8 hours after the fecal induction. We compared the proportion of species from unknown sources at the early time points with those at the late time points and observed significant differences in five out of six animals with Wilcoxon test (**Figure 2G**). By contrast, when we used 12-hour samples as the sink, we did not observe any trends. We then set the first three time points as T00, T02, and T04, and the last three time points as T09, T10, and T11, and we used Wilcoxon test to compare the two groups. We detected a significant difference between the early and late time points in only one animal (**Supplementary Figure 4**).

To validate the above findings ruling out low biomass contamination, we also analyzed two public datasets: a serially diluted mock community standard (Karstens et al., 2019) and a serially diluted *Salmonella bongori* culture (Salter et al., 2014) (**Supplementary Figure 5**). As expected, when we used the mock community standard as the sink, we observed a linear reduction in its proportion as the dilution factor increased. However, when we used the most dilute sample as the sink (mimicking low biomass at the initial time point), we observed no changes in the population. Similarly, for the *S. bongori* study (Salter et al., 2014), when we used 10^7^ cells or 10^3^ cells as the sink, we found that the populations were different from those in the blood microbiome. We hence concluded that the blood microbiome that we detected herein does not represent a contamination associated with sample preparation or “noise” (KitOme), even for the microbiome detected at the initial time point, which may be the “native blood microbiome” of each animal.

### Detection of Altered Blood Microbiome After Peritonitis Induction

**Figure 3:**
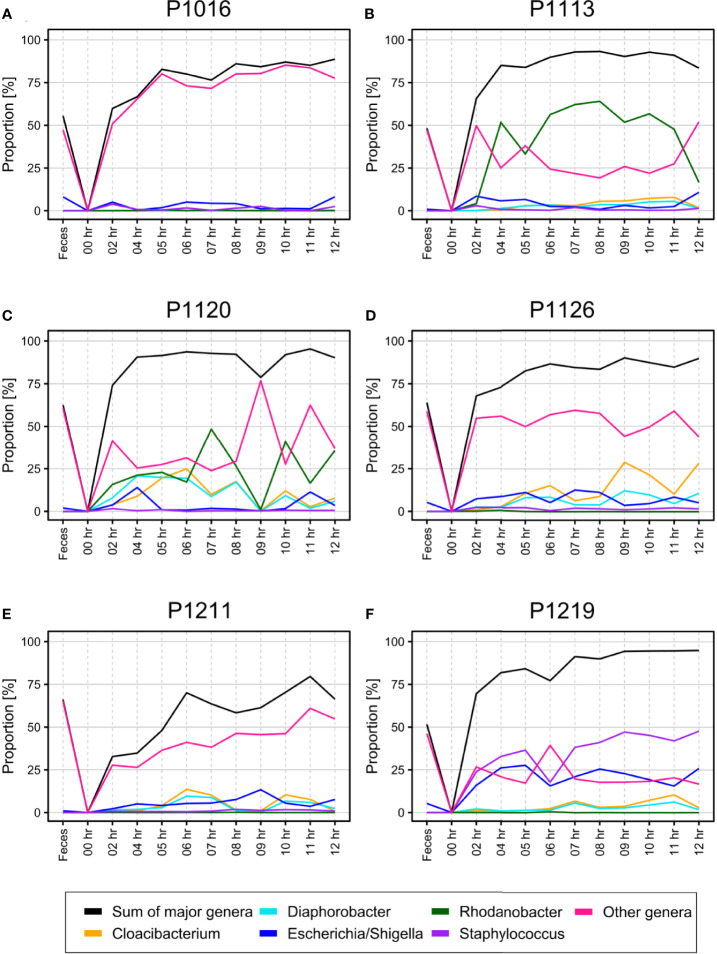
**(A–F)** Percentage of newly emerged operational taxonomic units (OTUs) from each phylum in the blood of pigs with fecal-induced peritonitis. The microbiome observed at the initial time point was discarded computationally from data for all other time points, and the proportion at each time point was re-calculated.

Although SourceTracker2 analysis revealed noticeable changes in the blood microbiome after peritonitis induction, the composition of the overall bacterial population was relatively consistent. We speculated that the pre-existing blood microbiome might mask small changes in the blood microbiome caused by peritonitis induction. To test this, we computationally discarded OTU clusters containing any OTUs observed at the initial time point, thus obtaining newly emerged OTUs for each sample. We noted gradual changes in bacterial populations as BSI progressed (**Figure 3**).

At the phylum level, the blood and fecal microbiomes harbored different amounts of *Proteobacteria* and *Firmicutes*. *Proteobacteria* was the most abundant phylum in the bloodstream of all animals but was not dominant in the feces. *Firmicutes* showed the opposite trend. We observed this difference regardless of computational filtering of the intrinsic microbiome population. Interestingly, *Bacteroidetes*, the representative gut microbiome phylum (Crespo-Piazuelo et al., 2019), was detected at all-time points, and its relative abundance gradually increased over time; this was not observed in the absence of the background blood microbiome (the light blue line in **Figure 3**). However, the composition of the blood microbiome did not change, even when we discarded the background populations (i.e., the microbiome detected before the fecal induction) from the analysis.

### Biological Pathways Enriched in the Blood Microbiome

**Figure 4:**
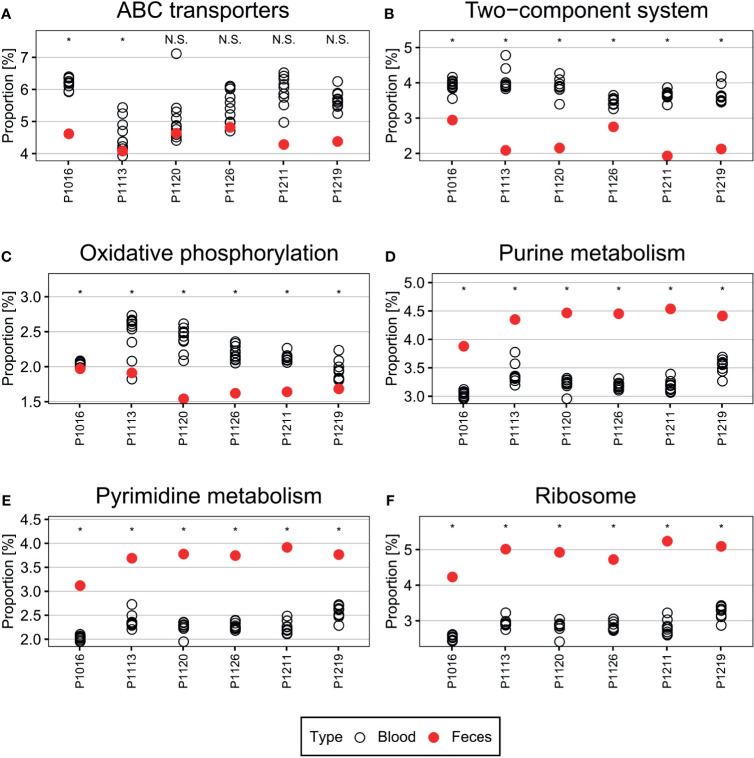
**(A–F)** Pathways differentially enriched in the blood microbiome. Pathways related to ABC transporters, two-component systems, and oxidative phosphorylation were over-represented in the blood microbiome compared with those in the fecal microbiome. By contrast, pathways related to central metabolism, such as purine and pyrimidine metabolism, and ribosome-related pathways, were under-represented. Wilcoxon test was used to check the significance of the enrichment and depletion of each pathway. *denotes the p-value of the Wilcoxon test is less than 0.05.

Genes and pathways enriched in a particular population are more relevant to microbiome function than bacterial composition (Gevers et al., 2014; Goodrich et al., 2014). In the porcine bacteremia model used in the current study, the blood is a unique environment for bacteria. It contains high levels of inflammatory cytokines and immune cells, with a unique composition of chemical compounds, such as lactic acid (Park et al., 2019). Therefore, we performed PICRUSt2 analysis to identify the putative functions of the blood microbiome (**Figure 4**). In the analysis presented above, we confirmed that new bacterial phyla have emerged into the bloodstream after peritonitis induction, and the populations of the native blood microbiome are notably different in each animal. So we utilized OTUs subtracting the native blood microbiome for this analysis to identify pathways essential for bacteria to survive in the bloodstream. Pathways related to ABC transporters, two-component systems, and oxidative phosphorylation were enriched in the blood microbiome. By contrast, pathways related to purine metabolism, pyrimidine metabolism, and ribosome expression were enriched to a lesser extent than observed in the feces. To validate these findings, we used Wilcoxon test to reveal pathways that are significantly enriched or depleted.

The ABC transporters and two-component systems constitute essential mechanisms that allow bacteria to appropriately respond to environmental signals (Messenger and Barclay, 1983; Levy, 2000; Gebhard, 2012; Mattos-Graner and Duncan, 2017). For example, some bacteria utilize ABC transporters to regulate acid–base balance and metal iron homeostasis (Messenger and Barclay, 1983; Gebhard, 2012). Furthermore, bacteria can use these transporters to defend themselves against antimicrobial peptides and proteins in the bloodstream (Levy, 2000; Gebhard, 2012). Two-component systems, each composed of a histidine kinase and a response regulator, are major bacterial signaling pathways that sense environmental cues (Mascher et al., 2006; Mattos-Graner and Duncan, 2017), such as pH (Gao and Lynn, 2005; Liu and Burne, 2009) and oxidative stress (Ortiz de Orué Lucana et al., 2012). They are also tightly linked to bacterial responses to the host immune system (Barrett and Hoch, 1998; Kawada-Matsuo and Komatsuzawa, 2017). Therefore, it is likely that cells in the microbiome would utilize these systems to survive.

Conversely, purine metabolism, pyrimidine metabolism, and ribosome expression were suppressed in the blood microbiome, which may limit cell proliferation and growth (Samant et al., 2008; Polymenis and Aramayo, 2015; Shaffer et al., 2017). Because the bloodstream is a harsh environment, cells therein may downregulate essential metabolic functions to survive. Simultaneously, reducing cell proliferation or maintaining low metabolic activity (e.g., dormancy) may enable bacteria to escape immune surveillance in the blood (Lennon and Jones, 2011; Rittershaus et al., 2013).

## Discussion

Here, we analyzed the blood microbiome in a bacteremia-induced porcine model. Microbiota transmission between organs, presumably *via* the bloodstream, has been reported in previous studies (Dominy et al., 2019; Massier et al., 2020; Sookoian et al., 2020). However, based on these studies, it is unclear how the blood microbiome is maintained because these studies provide only a “snapshot” view of a single point in time. By monitoring the blood microbiome over time, we here confirmed that each animal maintains a relatively consistent blood microbial population. Furthermore, by identifying potential pathways enriched in this population, we revealed that the bloodstream bacteria might have adapted to respond to the blood environment by using ABC transporters and two-component systems. On the other hand, the bacteria may not grow under these unfavorable conditions and, hence, pathways related to nucleotide biosynthesis may be suppressed under these conditions.

The biggest challenge to monitoring bacteria in the bloodstream is their low number compared with the microbiome at other body sites; such a low number means that even a minor contamination can have a major effect on the detection results (Eisenhofer et al., 2019; Karstens et al., 2019). To overcome this, we here selectively enriched bacteria from the blood using opsonin-coated MNPs (Kang et al., 2014) and then identified them by 16S rRNA gene sequencing. Nonetheless, it could have been difficult to distinguish the actual bacteria from bacterial DNA from the debris circulating in the bloodstream because the sequencing-based method is destructive (i.e., preparation for analysis involves bacterial cell lysis). Therefore, after capturing the bacteria with MNPs, we performed RNA-FISH targeting the common region within the bacterial 16S rRNA; this confirmed that bacteria were indeed present in the bloodstream, even before the induction of peritonitis (**Supplementary Figure 3**). Because of cell membrane integrity and a high abundance of ribosomal RNAs inside the cell, and because it is unlikely that DNA debris from as similar bacterial species can be observed over time, we concluded that the signal from the dead bacteria is not prominent in the analysis.

Dormant bacteria, which are metabolically suppressed and not immediately culturable, are quite common in the blood (Potgieter et al., 2015), and bacterial species that we identified by sequencing in the current study may have also been dormant. This may explain why we observed a discrepancy between the *in vitro* blood culture and the sequencing data. It is nonetheless surprising that similar (possibly dormant) bacterial populations were maintained in the blood over time, even after peritonitis induction, because the biological function of dormant bacteria is not well known. Further studies are required to understand their roles in the bloodstream.

Another common source of contamination in blood microbiota studies is the skin microbiome. According to one study, *Firmicutes* (55.6% of relative abundance), *Bacteroidetes* (20.8%), *Actinobacteria* (13.3%), and *Proteobacteria* (5.1%) are representative resident phyla in the porcine skin microbiota (McIntyre et al., 2016). Among them, *Staphylococcus* is the dominant genus within the skin microbiota of animals (Kloos et al., 1976) and humans (Byrd et al., 2018; O’Sullivan et al., 2019). If contamination with the skin microbiome had occurred, we should have observed these bacteria consistently, even in blood culture, because we sampled the blood *via* a catheter. However, these bacteria were not a major component of the blood microbiome in the current study, except one case (P1219), which showed a moderate amount of *Firmicutes* (**Figure 1**). Considering the above, we concluded that the skin microbiome contamination was reasonably controlled to analyze the blood microbiome in the current study.

The bacteremia model used herein yielded detectable bacteria in the blood after the induction (confirmed by culturing) (Park et al., 2019). We, therefore, used these samples as a “positive control” to detect the blood microbiome. We observed that the composition of the blood microbiome changed gradually after peritonitis induction by autologous feces, with an over-representation of *Bacteroidetes* from the gut microbiota (Crespo-Piazuelo et al., 2019) slightly increasing over time (**Figure 3**). The gut microbiome can enter the bloodstream when the host is immunocompromised (Taur and Pamer, 2013). Hence, the observed over-representation could indicate a septic symptom of peritonitis. When we systematically traced the microbiota source, we found that new populations were introduced gradually into the bloodstream 4–6 h after the peritonitis induction (**Figure 2**), mirroring the clinical symptoms, such as increased cytokine production (**Figure 6**). However, it is not clear whether these newly introduced bacteria induced the septic symptoms or entered the blood because of sepsis.

**Figure 5:**
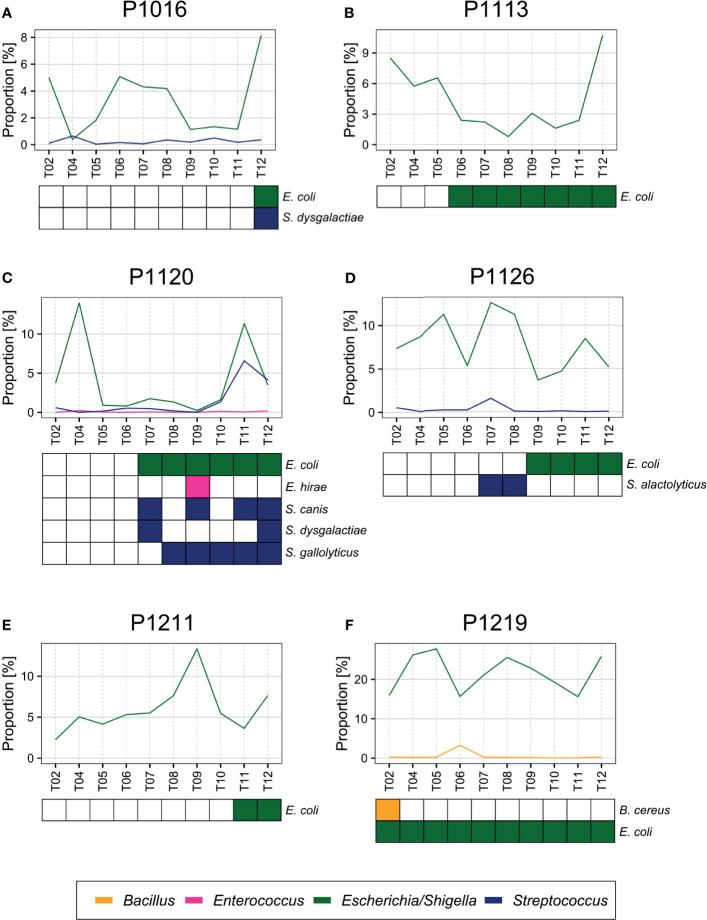
**(A–F)** Bacteria detected by blood culture. *E. coli* and other species (*Streptococcus dysgalactiae*, *Enterococcus hirae, Streptococcus alactolyticus*, *Streptococcus gallolyticus*, and *Bacillus cereus*) confirmed previously by blood culture (Park et al., 2019) were also detected by blood microbiome sequencing from each sample.

Surprisingly, the bacterial species identified by standard clinical laboratory culture testing were not the dominant species identified by culture-free bacterial population analysis, even though we observed a moderate association for the species and the time of detection (**Figure 5**). We speculate that culturable bacteria comprise only a small portion of the total blood microbiota. Further, the blood microbiome may enact a homeostatic mechanism that maintains the community members. The composition of the commensal microbiome is preserved upon exposure to exogenous bacteria (Littman and Pamer, 2011; Kamada et al., 2013; Khan and Shekhar, 2019), and the blood microbiome may operate a similar protective mechanism. Because the blood microbiome may not be metabolically active relative to other commensal bacteria, further study is required to examine this possibility.

The porcine peritonitis model used herein provides a unique opportunity to study the blood microbiome. Because of the large body size of pigs, the dynamics of the blood microbiome can be analyzed over time by multiple sampling, which is challenging when using a small animal model, such as a mouse. Although the animals used in the current study were not raised in an aseptic environment, the multiple sampling approach and computational methods for population comparisons used herein made it possible to comprehensively characterize the blood microbiota. Hence, this model may provide an excellent platform for developing new diagnostic techniques to detect BSI (Grumaz et al., 2016; Wanda et al., 2017; Horiba et al., 2018).

As reported in our previous study (Park et al., 2019), of the 82 bacterial species identified by blood culture, 83% were *Escherichia coli* (57.3%) or *Streptococcus* (25.7%) species, which were also identified by sequencing (**Figure 5**). For example, we identified *E. coli* and *Streptococcus dysgalactiae* in animals P1016 and P1120 at the late stages after the peritonitis induction, and we observed a significant increase in their relative abundance at the time point that coincided with blood culture positivity. In animal P1126, we detected *Streptococcus alactolyticus* only 7–8 h after the induction, and we detected *E. coli* at a later time point. Similarly, we observed a slight increase in the relative *Streptococcus* abundance 7–8 h after the induction in animal P1120. Moreover, we detected *E. coli* in animal P1219 at an early stage after the induction, and the sequencing data supported a high proportion of *E. coli* throughout the experiment of P1219.

On the other hand, based on the microbiome sequencing data, bacterial species confirmed by blood culture were not the most abundant in the bloodstream, even 12 h after the peritonitis induction. This could be attributed to that the sequencing might have detected bacterial DNA contained in white blood cells (Thwaites and Gant, 2011) or cell-free bacterial DNA (Wanda et al., 2017; Camargo et al., 2020). Although microbial DNA can induce inflammation and other host responses, it is a false positive in the analysis of the blood microbiome. Another explanation may be dormant bacteria, which cannot be cultured under test conditions (Lennon and Jones, 2011; Rittershaus et al., 2013; Potgieter et al., 2015). Because dormant bacteria can become reactivated depending on the environmental conditions, they can contribute to the blood microbiome function even if they are not immediately culturable. Further investigation is required to explain these discrepancies.

**Figure 6:**
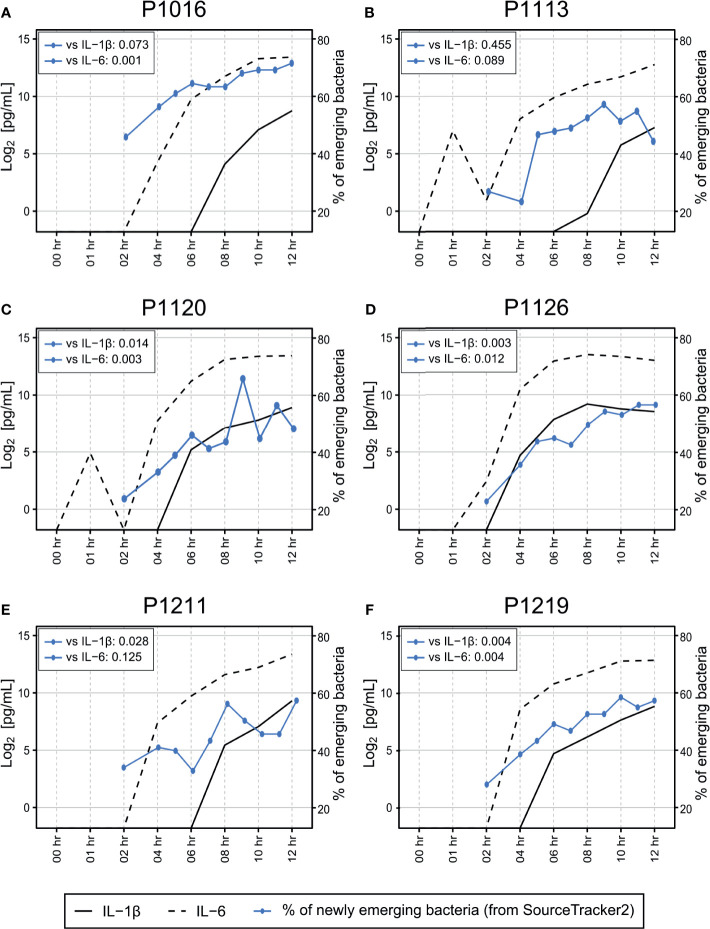
**(A–F)** Association between cytokine induction and introduction of new bacterial species into the bloodstream. Measurement of IL-1β and IL-6 levels in the blood revealed that most animals began to develop septic responses between 4 and 8 h after fecal induction. The proportion of newly emerging bacteria was determined by SourceTracker2 (**Figure 2**) by comparing the altered microbiome with the microbiome detected before peritonitis induction. The original cytokine data were published previously (Park et al., 2019). Pearson correlation coefficient p-value was calculated to verify the correlation between the newly emerging bacteria and cytokine markers.

Previously, we also reported that all animals showed symptoms relevant to sepsis, which developed gradually 5–6 h after the fecal induction (Park et al., 2019). Here, in addition to comparison with the blood culture result, we compared the emergence time points of the altered blood microbiome with those of pro-inflammatory host responses. Using SourceTracker2 analysis, we found that the levels of proinflammatory cytokines (interleukin (IL)-1β and IL-6) in the blood increased as the new microbiome emerged in the blood (**Figure 6**). We calculated the Pearson correlation coefficient p-value for those two parameters (the newly emerging bacteria and IL-1β or IL-6), and observed significant correlations between those for all animals except for P1113. Although we cannot at this point conclude whether the new blood microbiome plays a role in developing a septic symptom in the porcine peritonitis model induced by fecal inoculation, the data indicate some involvement of the blood microbiome.

Here, we reported changes in the composition of the blood microbiome in a porcine bacteremia model. By analyzing the blood microbiome in the same individual over time, we showed that the bacterial population remains relatively consistent, even after peritonitis induction. However, at the same time, we found that new bacterial populations entered the bloodstream, with the dynamic patterns similar to those observed during a physiological response to BSI (e.g., cytokine level increase). Further, by analyzing population-enriched pathways, we confirmed that sensing mechanisms, such as ABC transporters and two-component systems, are upregulated in the blood microbiome. Conversely, central nucleotide metabolism, essential for cell proliferation and growth, was suppressed in these bacteria, which probably helps the blood microbiome to survive the harsh bloodstream environment and escape immune surveillance. Finally, the current study indicates that further investigations of the blood microbiome are required to improve the current diagnostic approaches for BSI.

## Data Availability Statement

The datasets presented in this study can be found in the European Nucleotide Archive (ENA) under the accession ID PRJEB39083.

## Ethics Statement

The animal study was reviewed and approved by Seoul National University Bundang Hospital IACUC (BA1804-246/040-01).

## Author Contributions

HH performed the microbiome experiments and data analysis, assisted by SY and HSK. MSL prepared the blood microbiome samples and performed the FISH experiment. IP performed the animal experiments, assisted by D-HJ and SK. HK, JHK, JHL, and TK conceived the study and designed the experiments. JK, JL, and TK analyzed the data. All authors contributed to the article and approved the submitted version.

## Funding

This research was supported by grants from the National Research Foundation of Korea (2017M3A9E2062138 to TK, 2017M3A9E2062136 to JHK, 2017M3A9E2062210 to JHL); and by the Basic Science Research Program, through the National Research Foundation of Korea, funded by the Ministry of Education (2018R1A6A1A03025810 to TK), and partially by the Future-leading Project Research Fund of UNIST (1.210034.01 to TK and JHK).

## Conflict of Interest

The authors declare that the research was conducted in the absence of any commercial or financial relationships that could be construed as a potential conflict of interest.

## Publisher’s Note

All claims expressed in this article are solely those of the authors and do not necessarily represent those of their affiliated organizations, or those of the publisher, the editors and the reviewers. Any product that may be evaluated in this article, or claim that may be made by its manufacturer, is not guaranteed or endorsed by the publisher.

## Abbreviations

BSI, bloodstream infection; IL, interleukin; KEGG, Kyoto Encyclopedia of Genes and Genomes; MNP, mannose-binding lectin(MBL)-coated magnetic nanoparticles; OTU, operational taxonomic unit; PICRUSt, Phylogenetic Investigation of Communities by Reconstruction of Unobserved States.
