# Supplementary material for: Analysis of Porcine Model of Fecal-Induced Peritonitis Reveals the Tropism of Blood Microbiome
Source: Front Cell Infect Microbiol. 2021 Aug 30;11:676650. doi: 10.3389/fcimb.2021.676650 (PMC8435847; doi:10.3389/fcimb.2021.676650)
Supplement: Supplementary file 1 [file DataSheet_1.pdf]

Supplementary Material

1 Supplementary Data

Supplementary Data 1. OTU tables clustered using VSEARCH after the genus or phylum assignment with the RDP classifier

Supplementary Data 2. Phylum tables for the identified blood microbiome for each animal

Supplementary Data 3. Genus tables for the identified blood microbiome for each animal

Supplementary Data 4. SourceTracker2 analysis of the proportion of novel species not present at the initial time point (T00)

2 Supplementary Figures and Tables

2.1 Supplementary Figures

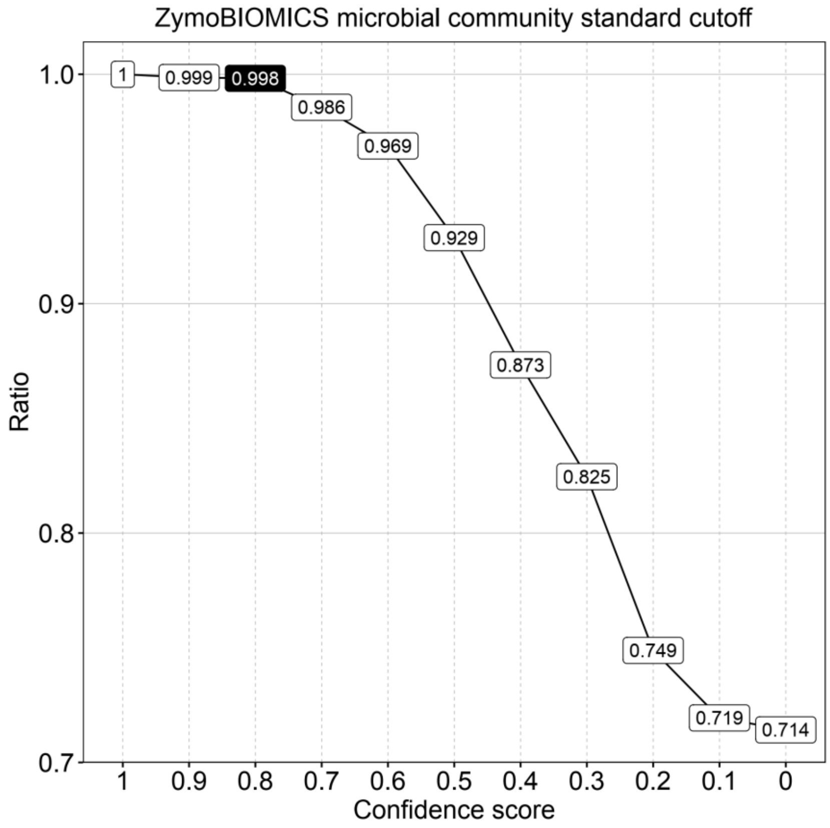

Supplementary Figure 1. The cut-off value of the RDP classifier, based on a standard microbiome dataset. The V34 amplicons of the 16S rRNA gene from the Zymo Microbiome Standard sample, which contains eight different bacterial genomes, were sequenced. The data were assigned to taxonomies using the RDP classifier (version 2.11), and the number of true-positive reads (numbers inside the box) was evaluated. Based on this analysis, the RDP classifier confidence score was set as 0.8 (cut-off for analysis), which assigned 99.8% reads correctly to their target.

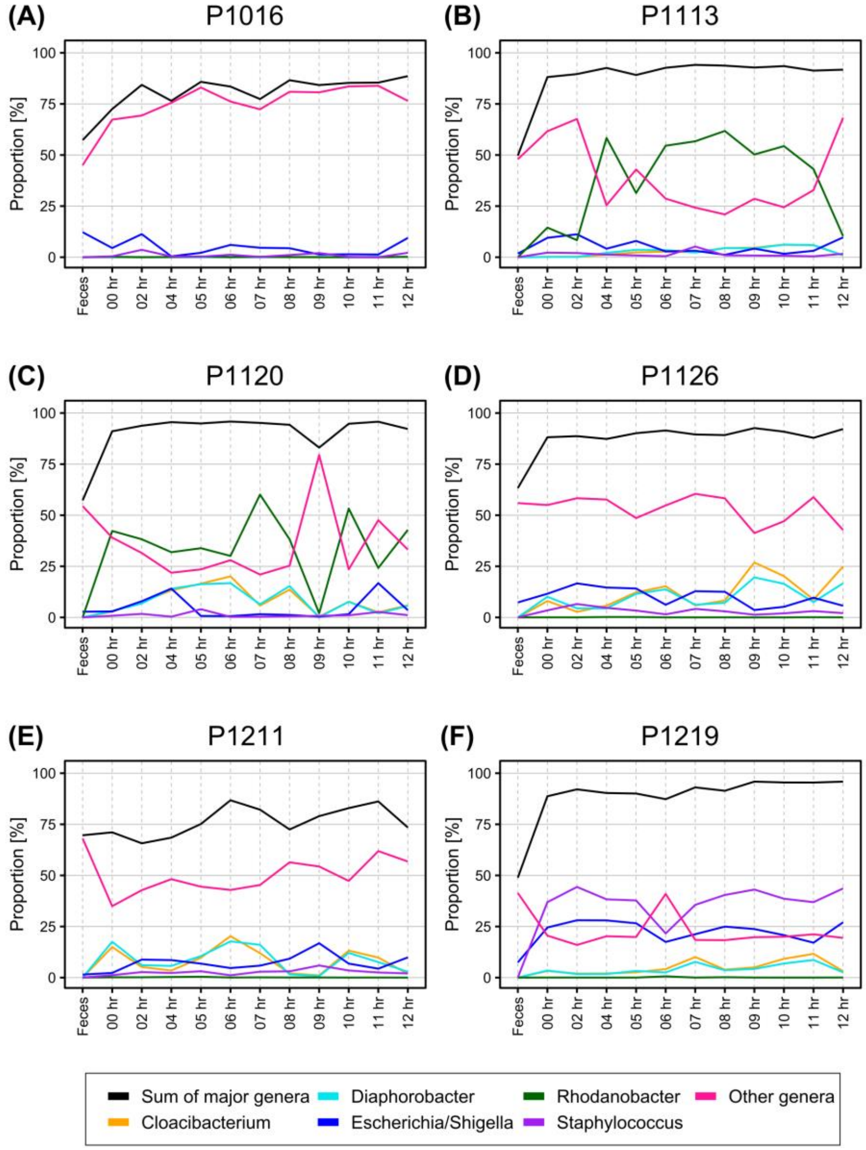

Supplementary Figure 2. Percentage of operational taxonomic units (OTUs) for each genus identified in the blood of pigs with fecal-induced peritonitis.

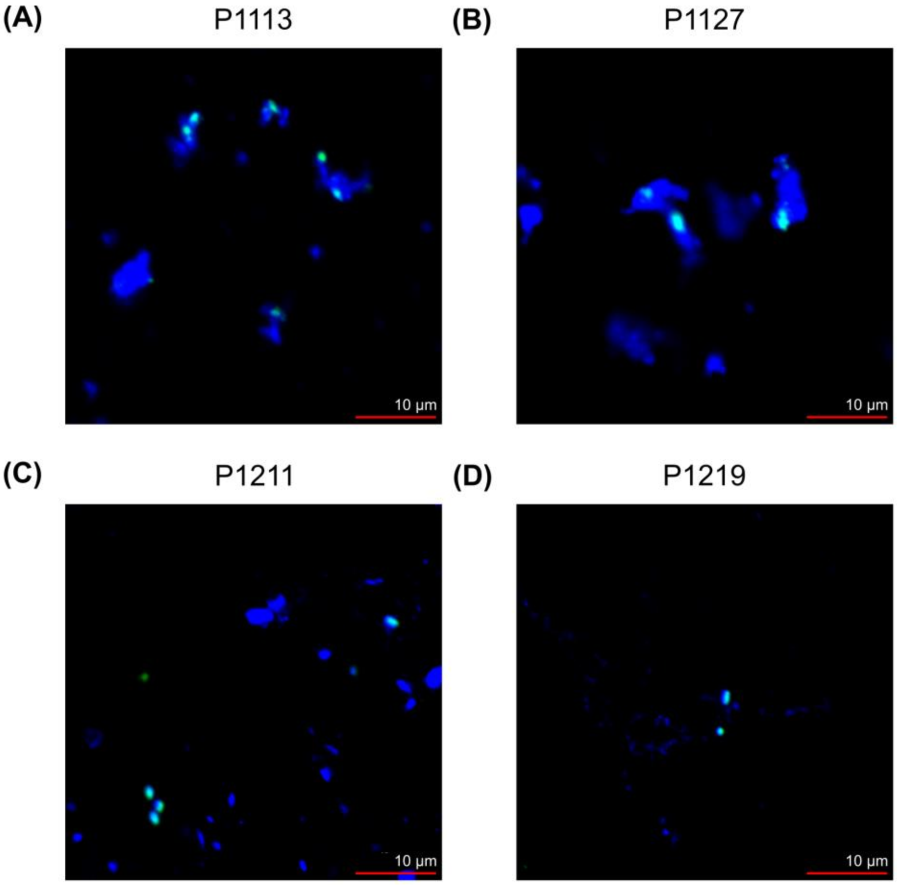

Supplementary Figure 3. RNA-FISH analysis before fecal induction. Because blood cells are also captured, DAPI signals (blue) covering a large area were also observed. Universal probe which targets the conserved region of 16S rRNA, was used to detect bacteria in the bloodstream. The probe signal (green) overlapped with the blue signal (cyan in merged images), indicating the blood microbiome.

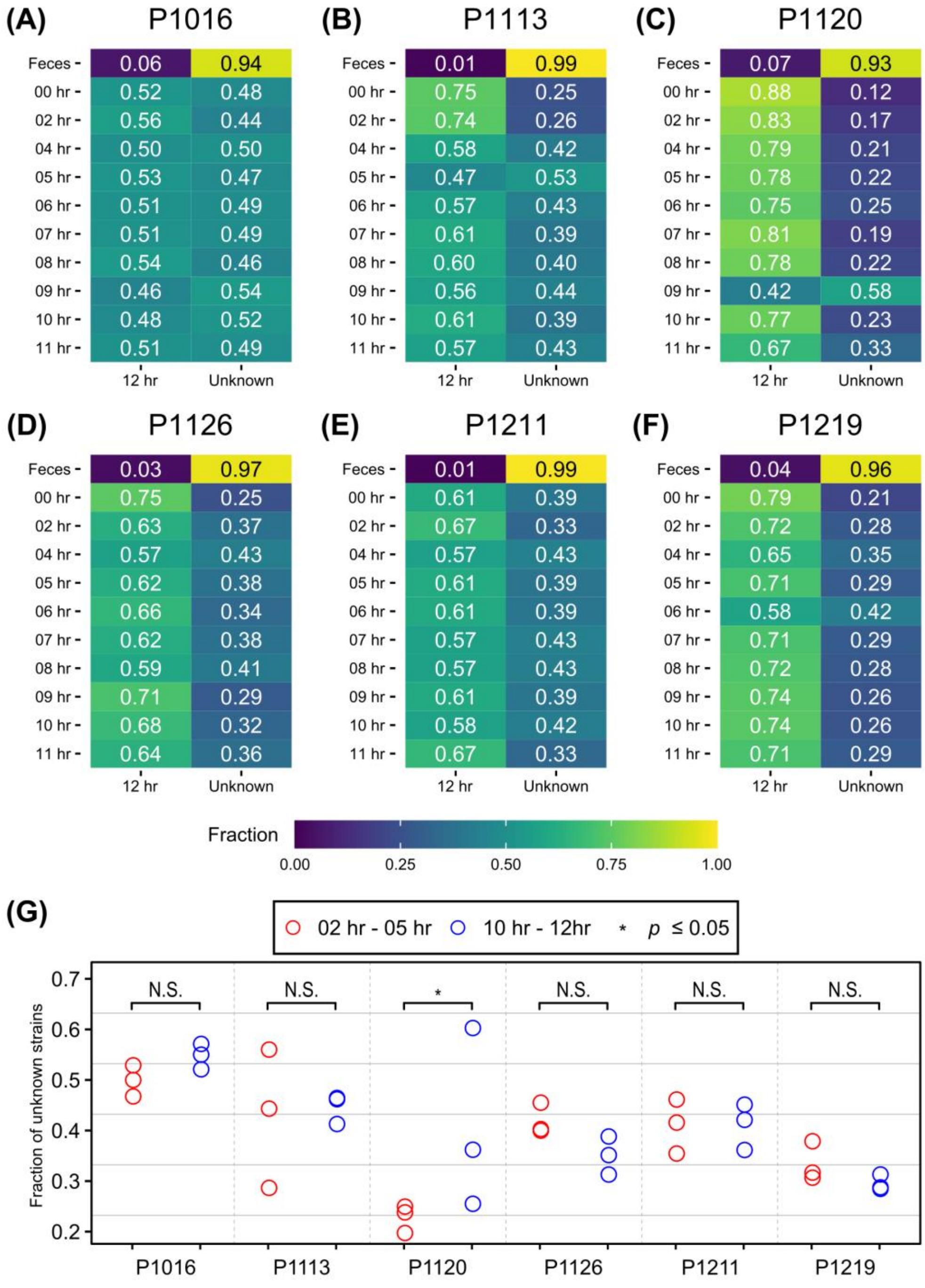

Supplementary Figure 4. SourceTracker2 analysis showing random patterns when the microbiome observed at the last time point (12 h after peritonitis induction) was used as the source. Almost all samples contained > 50% of the bacterial species identified at the end of the experiment, but those numbers did not change during peritonitis induction. Hence, no time-related changes were observed (compared with Figure 2).

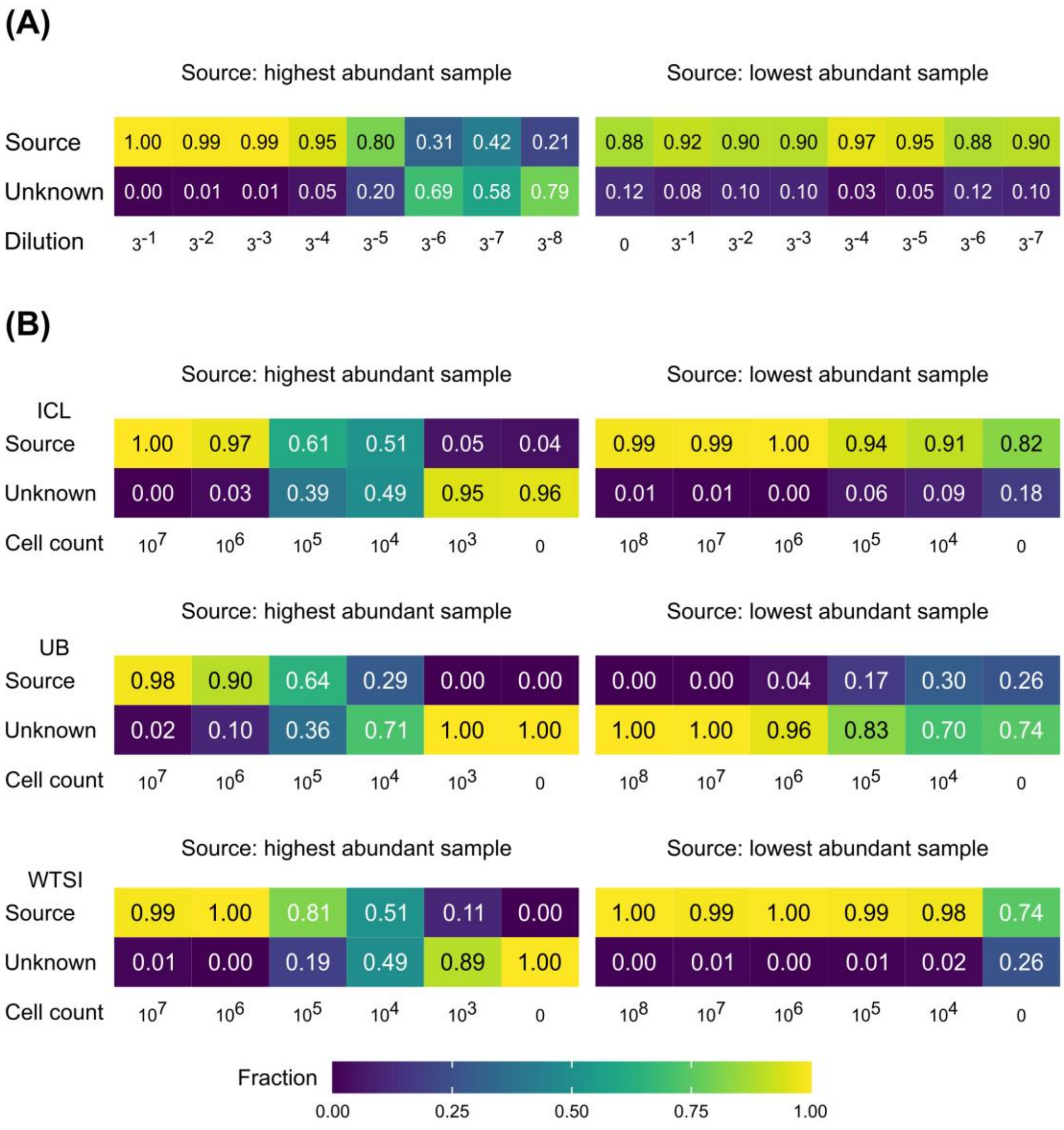

Supplementary Figure 5. SourceTracker2 analysis showing distinctive patterns of contamination associated with low cell numbers in samples. Serial dilution of the sample with the highest bacterial abundance showed a gradual decrease in the proportion of the original population (left) in all samples. However, when the low abundance sample (3^{-8} dilution for [a] or 10^{3} cells for [b]) was used as the source, no pattern was observed.

2.2 Supplementary Tables
